# Therapeutic communication improves patient comfort during venipuncture in children: a single-blinded intervention study

**DOI:** 10.1007/s00431-023-05036-7

**Published:** 2023-06-17

**Authors:** Lonneke A. M. Aarts, Geert-Jan van Geffen, Eva A. L. Smedema, Rosanne M. Smits

**Affiliations:** 1 Department of Pediatrics, Amalia Children’s Hospital, Radboud University Medical Center, Nijmegen, the Netherlands; 2 Department of Anesthesiology, Radboud University Medical Center, Nijmegen, The Netherlands; 3 Department of Medical Psychology, Amalia Children’s Hospital, Radboud University Medical Center, Nijmegen, the Netherlands

**Keywords:** Therapeutic communication, Venipuncture, Neurodivergent, Pain, Anxiety, Patient comfort, Parental anxiety

## Abstract

The aim of this study was to examine whether therapeutic communication improves children’s comfort during venipuncture compared to standard communication. This study was registered in the Dutch trial register (NL8221), December 10, 2019. This single-blinded interventional study was carried out in an outpatient clinic of a tertiary hospital. Inclusion criteria were age between 5 and 18 years, use of topical anesthesia (EMLA) and sufficient understanding of the Dutch language. 105 children were included, 51 assigned to the standard communication group (SC group) and 54 patients to the therapeutic communication group (TC group). The primary outcome measure was self-reported pain based on the Faces Pain Scale Revised (FPS-R). Secondary outcome measures were observed pain (numeric rating scale (NRS)), self-reported/observed anxiety in child and parent (NRS), self-reported satisfaction in child, parent and medical personnel (NRS), and procedural time. No difference was found for self-reported pain. Self-reported and observed anxiety (by parents and medical personnel) was lower in the TC group (*p*-values ranged from *p* = 0.005 to *p* = 0.048). Procedural time was lower in the TC group (*p* = 0.011). Satisfaction level of medical personnel was higher in the TC group (*p* = 0.014).

*Conclusion*: TC during venipuncture did not result in lower self-reported pain. However, secondary outcomes (observed pain, anxiety and procedural time) were significantly improved in the TC group.

**Table:** 

**What is Known:** *• Medical procedures, especially needle related procedures, cause anxiety and fear in children and adults.* *• In adults communication techniques based on hypnosis are effective in reducing pain and anxiety during medical procedures.*	
**What is New:** *• Our study found that with a small change in communication technique (called therapeutic communication or TC), the comfort of children during venipuncture improves.* *• This improved comfort was mainly reflected by reduced anxiety scores and shorter procedural time. This makes TC suitable for the outpatient setting.*

**Supplementary Information:**

The online version contains supplementary material available at 10.1007/s00431-023-05036-7.

## Introduction

Many children feel anxious when they need to undergo a medical examination or intervention. In particular, needle related procedures cause anxiety and fear in children [1–6]. This leads to more pain and emotional distress [2–6]. Failure to adequately manage pain and anxiety may have immediate negative consequences such as unsuccessful medical procedures and lack of cooperation from a child, leading to increased procedural time and adverse physiological reactions [3, 4, 6]. More far-reaching effects are increased pain perception, diminished analgesic effectiveness with subsequent procedures, and avoidance of medical care, which may persist into adulthood [4, 6]. Besides negatively impacting the child, significant child pain and anxiety during needle procedures can be highly distressing and challenging for parents and healthcare providers as well [7, 8].

In adults, the use of communication techniques based on hypnosis can be used to reduce pain and anxiety [9, 10]. These methods are based on establishing instant rapport, avoiding negative suggestions and reframing using a script or guided imagination. These techniques can be integrated in everyday patient interaction and as a supplement on procedural sedation or perioperative care [9–11]. Non-pharmacological psychological interventions for pain have also been investigated in children in the review of Birnie [2]. Distraction, breathing, hypnosis and combined cognitive and behavioral strategies can reduce pain and distress during needle procedures. The results of this review, however, should be interpreted with caution due to lack of blinding of patients and of outcome assessors [2]. To further examine the effects of communication techniques on procedural pain and comfort in children, more research is warranted.

Children experience medical procedures within the context of their family [12]. This means that a child’s coping strategy and its expectations are related to that of its parents [12]. So, when examining the effects of an intervention on pain and distress of the child, it is of importance to also assess the parent experience of this intervention (e.g., by measuring proxy pain, and self-reported and observed anxiety). Several studies have addressed the influence of parental anxiety on the child’s pain experience [13–16]. For example, one of the studies showed that parent (pre)procedural anxiety increases the child’s anxiety during the procedure which ultimately caused more child’s pain [13]. Therefore, in this study we will investigate the influence of parental anxiety on the child’s pain experience.

This study assesses the effects of therapeutic communication (TC) versus standard communication (SC). TC is based on Comfort Talk^®^, the LAURS of hypnotic communication and the ''Lived in Imagination'' Technique in an outpatient setting [9–11, 17, 18]. The objective of this study is to examine whether patient-centric TC positively affects children’s comfort during venipuncture. The primary outcome of this study is self-reported pain by children. Secondary outcomes are observed pain (child and parent), anxiety (child and parent), satisfaction (child, parents and medical personnel) and procedural time. Also, a mediation analysis will be conducted to investigate whether parents’ anxiety increases children’s pain through children’s anxiety. Finally, a subgroup analysis will be performed to investigate if TC is effective in neurodivergent children (e.g., with attention-deficit-hyperactivity-disorder (ADHD) or with autism spectrum disorder (ASD). This, to gain more insight in this topic as a considerable amount of children in our tertiary hospital is neurodivergent and there is lack of empiric evidence on patient comfort and venipuncture in these patients [19].

## Methods and materials

### Study design

After ethical approval (Ethics Committee CMO Arnhem-Nijmegen, Number 2019-5488), this single-blinded intervention study was performed in an outpatient clinic in a tertiary care hospital in Nijmegen, the Netherlands. The study was registered in the Dutch Trial Register (NL8221). Data was collected between January 2020 and October 2020.

### Participants

Children who needed to undergo venipuncture at the outpatient clinic were asked to participate in this study. Inclusion criteria were age between 5 and 18 years, use of topical anesthesia on the presumed needle insertion site (EMLA, Aspen, The Netherlands), and the ability to communicate in Dutch language by child and parent or guardian. Written informed consent was obtained prior to the study. For children in the age of 5 to 12 years or children with intellectual disabilities, informed consent was provided by parent or guardian. For children aged 12 to 16 years, it was provided by the child and parent or guardian, and for children up to and including 16 years, it was provided by the child.

### Procedure

**Fig. 1:**
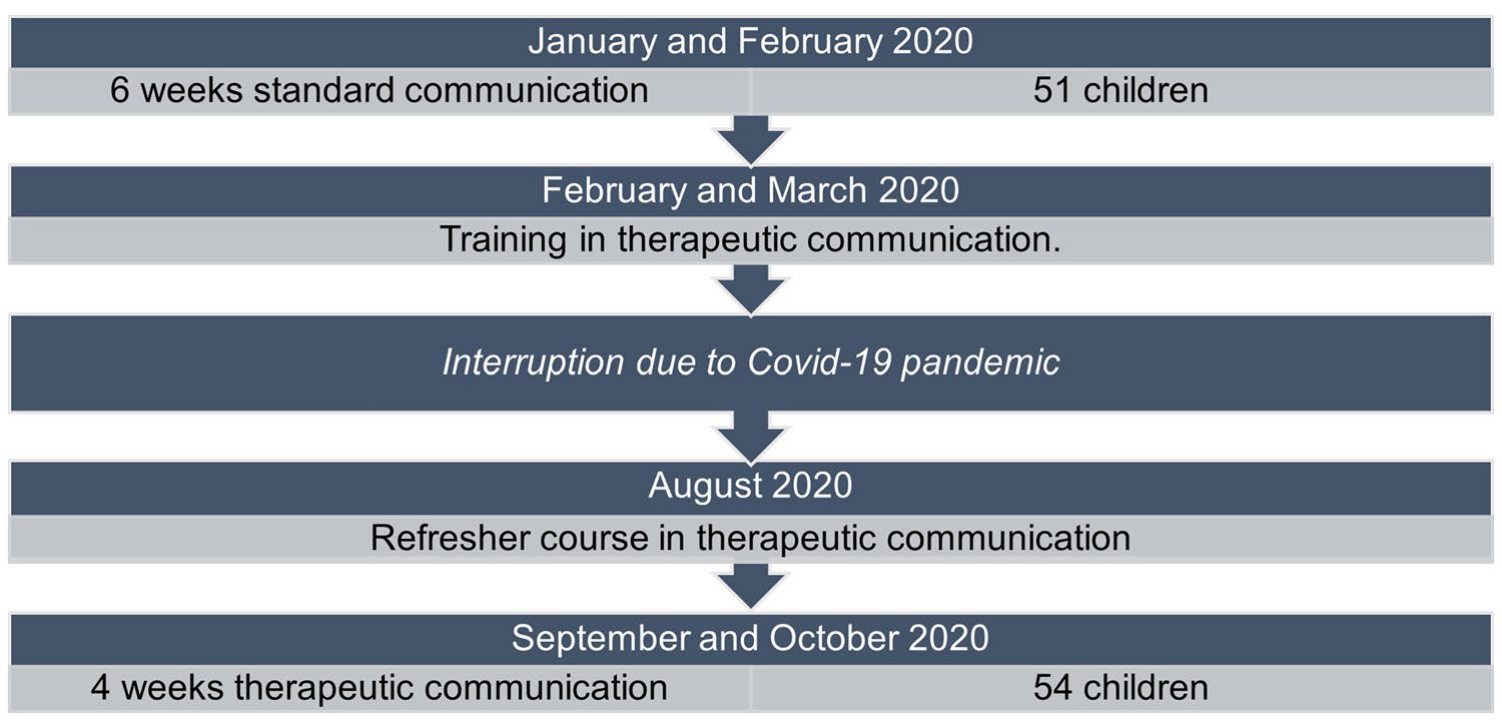
Flowchart of the study

The study consisted of two groups that were subsequently studied: a SC group and a TC group. The SC group was studied in January and February 2020, and the TC group in September and October 2020. For the SC group, blood sampling procedures were performed according to standardized protocol and baseline data were obtained. After having included 51 patients, all personnel was trained in patient-centered TC. The training consisted of an eight-hours classroom session in which theoretical lectures about the scientific background were interchanged with practical demonstrations, exercises, and role-playing. Medical personnel was trained in how to establish quick empathic contact by matching nonverbal and verbal behavior, avoidance of negative verbal suggestions, the use of hypnotic language and expression of positive expectations (Supplementary Table 1: an overview of therapeutic communication techniques applied in our study). In August 2020, a four-hours refresher course was organized because of an interruption due to COVID-19. The TC group ended in October 2020 when 54 children were included (Fig. 1).

## Measures

### Demographic data

Demographic data was obtained prior to venipuncture (e.g. age, gender, underlying disease, amount of previous venipunctures and the presence of neurodivergent disorders (e.g. ADHD, ASD). To measure anxiety the child’s trait level of anxiety and the parents’ trait and state level of anxiety were obtained by the Spielberger State-Trait Anxiety questionnaire for children (STAI-C) and the Dutch version of the State-Trait Anxiety Inventory (STAI-Y), named ZBV for the parents [20].

### Primary outcome measure

#### Self-reported pain

Self-reported pain was measured directly after venipuncture with the Faces Pain Scale-Revised (FPS-R) [21]. The FPS-R has been recommended for use following procedure-related pain in clinical trials for children of four years and older [21–23].

### Secondary outcome measures

#### Observed pain

Directly after the venipuncture parents or guardians and medical professionals scored the observed pain of the child on a NRS (Numeric Rating Scale; 0 = no pain, 10 = worst pain ever).

#### Anxiety during venipuncture

Children 8 years or older reported their anxiety levels during venipuncture on a NRS (0 = no anxiety, 10 = worst anxiety ever). Parents or guardians and medical professionals scored the observed anxiety of the child and anxiety of the parent on the same NRS.

#### Satisfaction

Children 8 years or older, their parents or guardians and medical personnel reported their satisfaction about the procedure on a NRS (0 = unsatisfied, 10 = very satisfied).

#### Procedural time

The time the child spent in the room during venipuncture was measured in minutes.

### Statistical analysis

For data analysis, IBM SPSS Statistics 26 was used. A power analysis was performed to determine the sample size that was needed to provide a 95% probability of detecting differences between groups. One face (i.e., 2 points, translating to an effect size of *d* = 0.8) on the FPS-R scale was assumed to be a clinically significant difference as proposed by Tsze et al. for the 7-point Faces Pain Scale [24]. The sample size was calculated with G*Power 3.1 and the required number to include was 42 in the SC group and 42 in the TC group in order to detect a statistically significant difference (p < 0.05).

 Baseline group differences for demographic characteristics were determined with independent sample *t*-tests for continuous outcomes (age and anxiety) and with Chi-square tests for categorical outcomes (sex, psychiatric disorder, involvement of child life specialist, amount of venipunctures, use of calming medication).

 To answer the primary research aim, namely to assess whether self-reported pain was lower after TC than SC, an independent sample *t*-test was performed to compare self-reported pain between the SC and the TC group. For the secondary aim of this study, namely to detect group differences between the SC and TC group for observed pain scores (by researcher, parent and medical personnel), anxiety scores (of the child and parent), satisfaction scores (of the child, parents and medical personnel), and time in the room, independent sample *t*-tests were performed. To answer the third research question, two mediation analyses were conducted for both groups to investigate whether parent procedural anxiety influences child procedural anxiety, which in turn, affects child’s procedural pain. Both analyses included parent self-reported procedural anxiety, child-reported procedural anxiety, and child-reported procedural pain. The Preacher and Hayes mediation macro was used to estimate indirect effects [25]. Finally, a subgroup analysis of neurodivergent children was performed with two-way analysis of variance (ANOVA), to investigate a possible interaction effect on anxiety between the of SC or TC group and having an ADHD/ASD diagnosis.

## Results

### Sample characteristics

In total, 105 children (range 5–17 years old) participated in the study. Demographic data are described in Table 1.

**Table 1:** Sample characteristics

**Sample characteristics**	**TC group** **(n = 54)**	**SC group (n = 51)**	***t***	***p***
Age	9.0 (3.4)^a^	9.8 (2.9)^a^	1.17	0.245
Anxiety child (STAI* trait)	31.3 (8.3)^a,b^	34.6 (10.1)^a^	1.83	0.071
Anxiety parent (STAI trait)	33.4 (8.7)^a,b^	33.4 (7.9)^a^	0.01	0.994
Anxiety parent (STAI state)	27.8 (6.2)^a,c^	29.4 (7.2)^a^	1.21	0.229
			***X***^**2**^	***p***
Sex – (male, female: n, %)	34 (63.0%), 20 (37.0%)	29 (56.9%), 22 (43.1%)	0.41	0.555
Neurodivergency -				
No (n,%) Yes (n, %)	39 (72.2%) 15 (27.8%)	36 (70.6%) 15 (29.4%)	0*.*03	1.000
- ADHD - ASS - Other	- 7 (13.0%) - 7 (13.0%) - 6 (11.8%)	- 5 (9.8%) - 6 (11.8%) - 7 (13.0%)	0.26 0.04 0.04	0.762 1.000 1.000
Pedagogic worker involved (n, %)				
No (n, %) Yes (n, %)	46 (85.2%) 8 (14.8%)	43 (84.3%) 8 (15.7%)	0.02	1.000
Amount of venipunctures in medical history			4.31	0.30
0 (n, %) 1 (n, %) 1–4 (n, %) > 5 (n, %)	5 (9.3%) 2 (3.7%) 4 (7.4%) 43 (79.6%)	1 (2.0%) 3 (5.9%) 8 (15.7%) 39 (76.5%)		
Calming medication^d^				
No (n, %) Yes (n, %)	49 (90.7%) 5 (9.3%)	46 (90.2%) 5 (9.8%)	0.01	1.000

*STAI State-Trait Anxiety Inventory

^a^Mean (SD)

^b^n = 50

^c^n = 51

^d^Medication used were methylphenidate (6 patients, 3 in both groups), dexamphetamin (1 patient in SC group), haloperidol (1 patient in TC group), risperidone (1 patient in TC group), aripripazole (1 patient in the TC group)

There were no differences in sample characteristics between the SC and TC group. Anxiety levels (measured with the STAI trait) did not differ between the SC and TC group. Outcome measures for the statistical analyses were checked for corresponding assumptions. Data of self-reported and observed pain scores were not normally distributed, and were therefore also analyzed with non-parametric tests (Mann–Whitney U test). Because this yielded no difference in results, all outcomes reported below are from parametric tests (independent sample *t*-tests, two-way ANOVA and regression analysis). See Table 2 for an overview of outcomes of group comparisons.

**Table 2:** Outcomes of group comparisons

**Outcomes**	**TC group (n = 54)**			**SC group (n = 51)**			***t***	***p***	***Cohen’s d***
	***M***	***SD***	***95%CI***	***M***	***SD***	***95%CI***			
Self-reported pain									
FPS-R^a^	1.7	2.2	[1.1, 2.3]	1.7	2.6	[0.9, 2.4]	0.42	.967	0.00
Observed pain									
NRS^b^ by parent	1.2	1.6	[0.7, 1.6]	1.6	2.1	[1.0, 2.2]	1.20	.232	0.48
NRS by nurse	0.8	1.8	[0.4, 1.3]	1.2	2.1	[0.7, 1.8]	1.07	.285	0.21
Anxiety									
NRS by child^c^	2.6	2.7	[1.6, 3.5]	4.0	3.0	[3.0, 5.0]	2.05	.045*	0.40
NRS child by parent	3.2	3.2	[2.3, 4.1]	4.7	3.3	[3.8, 5.6]	2.36	.020*	0.49
NRS child by nurse	2.7	2.8	[1.9, 3.5]	4.1	3.1	[3.3, 5.0]	2.47	.015*	0.46
NRS parent by parent	0.5	1.1	[0.2, 08]	1.3	1.9	[0.7, 1.8]	2.55	.013*	0.50
NRS parent by nurse	0.5	1.1	[0.2, 0.8]	1.0	1.6	[0.5, 1.4]	1.56	.122	0.30
Satisfaction									
NRS by child^c^	8.6	3.1	[7.5, 9.7]	8.8	1.8	[8.2, 9.4]	0.32	.750	0.07
NRS by parent	9.2	1.4	[8.8, 9.6]	9.4	0.9	[9.1, 9.6]	0.64	.524	0.13
NRS by nurse	9.2	1.1	[8.9, 9.5]	8.5	1.6	[8.0, 9.0]	2.65	.009*	0.52
Time in the room	6.0	2.2	[5.4, 6.6]	8.6	6.8	[6.7, 10.5]	2.53	.014*	0.50

*p < 0.05

^a^Faces Pain scale Revised

^b^Numeric Rating Scale

^c^Children of 7 years and older (n = 33 TC group, n = 38 SC group)

### Primary outcome measure

#### Self-reported pain by children

**Fig. 2:**
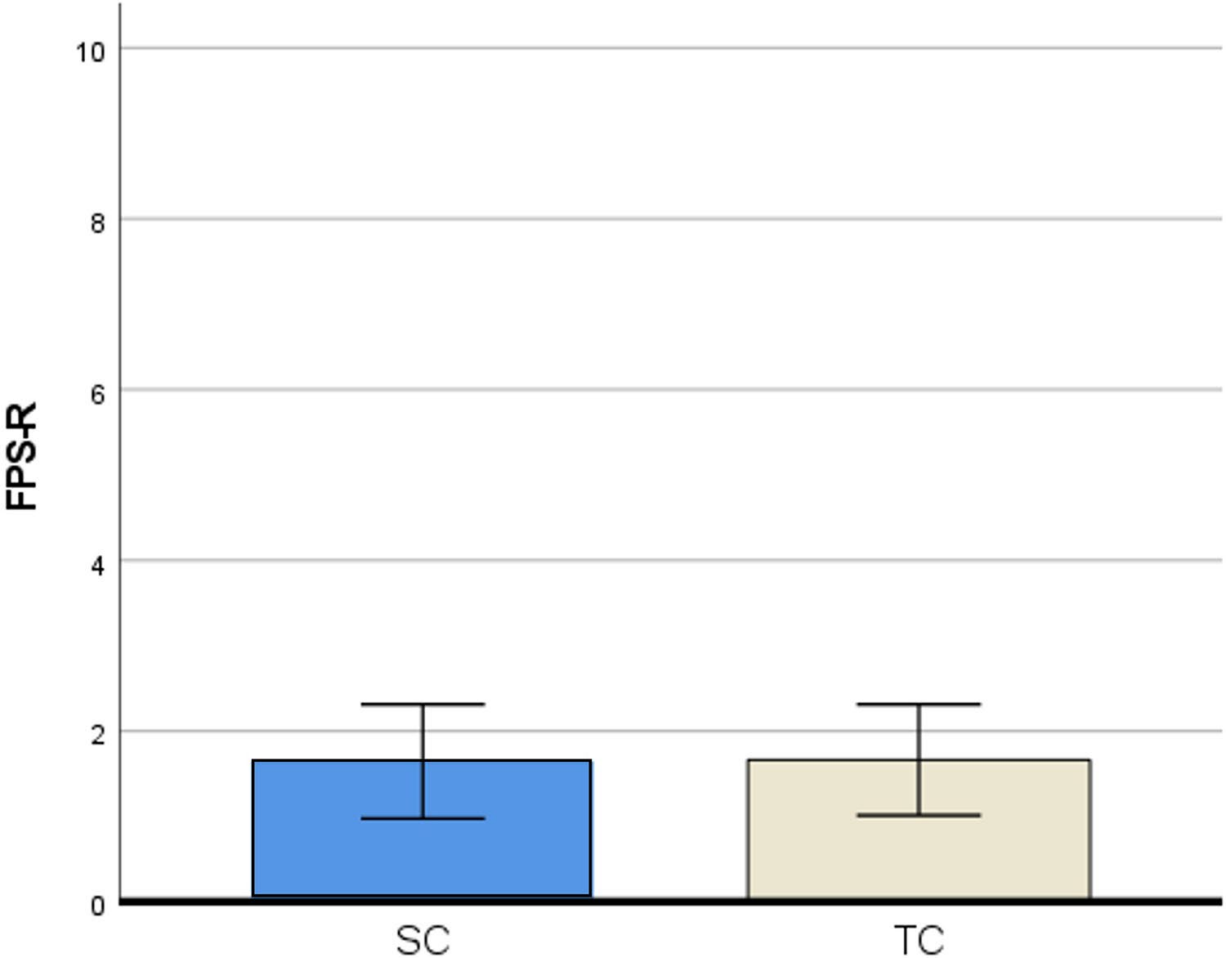
Child pain outcomes. *SC* = *Standard communication, TC* = *Therapeutic communication, FPS-R* = *Faces pain scale Revised*

There was no difference in self-reported pain by children between the TC group (*M* = 1.7, *SD* = 2.2) and SC group (*M* = 1.7, *SD* = 2.6), *t*(103) = 0.42, *p* = .967 (Fig. 2).

### Secondary outcome measures

#### Observed pain

NRS pain scores observed by parents and by medical personnel did not significantly differ between the TC and SC group (Table 2).

#### Anxiety during venipuncture

**Fig. 3:**
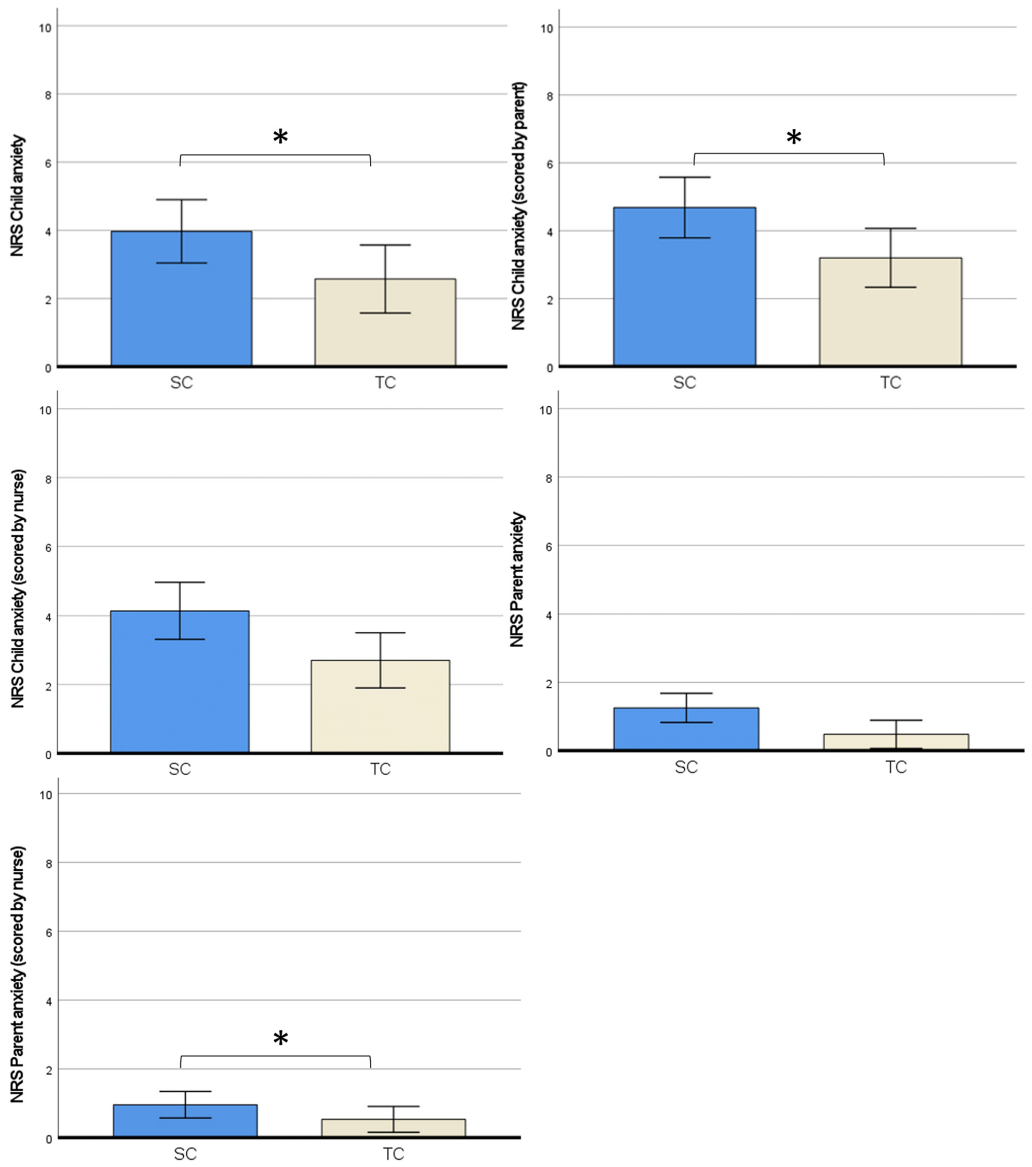
Anxiety outcomes. *SC* = *Standard communication, TC* = *Therapeutic communication, NRS* = Numeric rating scale. 95% Error bars, * < *.05*

For anxiety scores scored by the child, we found that children in the therapeutic communication group reported lower anxiety *t*(69) = 2.05, *p* = 0.45. For the other anxiety outcomes, we found that anxiety scores of the child were significantly lower in the TC group when scored by the parent *t*(103) = 2.36, *p* = .020, and by medical personnel *t*(103) = 2.47, *p* = .015. Anxiety scores of the parent were lower in the TC group when scored by the parents themselves *t*(103) = 2.55, *p* = .013, but not when scored by medical personnel (Fig. 3).

#### Satisfaction

**Fig. 4:**
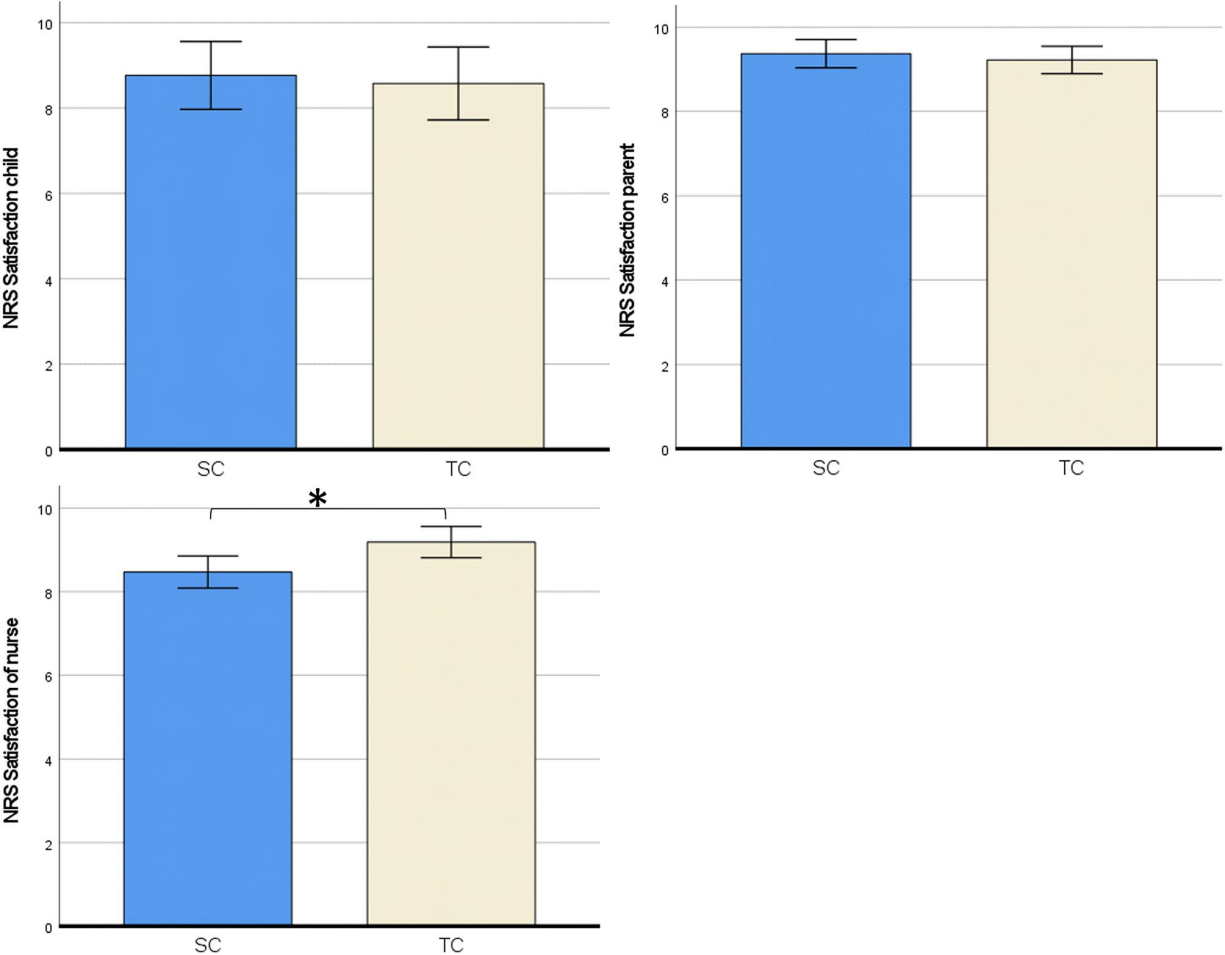
Satisfaction outcomes. *SC* = *Standard communication, TC* = *Therapeutic communication, NRS* = Numeric rating scale. 95% Error bars, * < *.05*

There were no group differences in satisfaction for parents and children (Table 2). Medical personnel reported that they were more satisfied after TC than after SC *t*(102) = 2.65, *p* = 0.009 (Fig. 4).

#### Procedural time

**Fig. 5:**
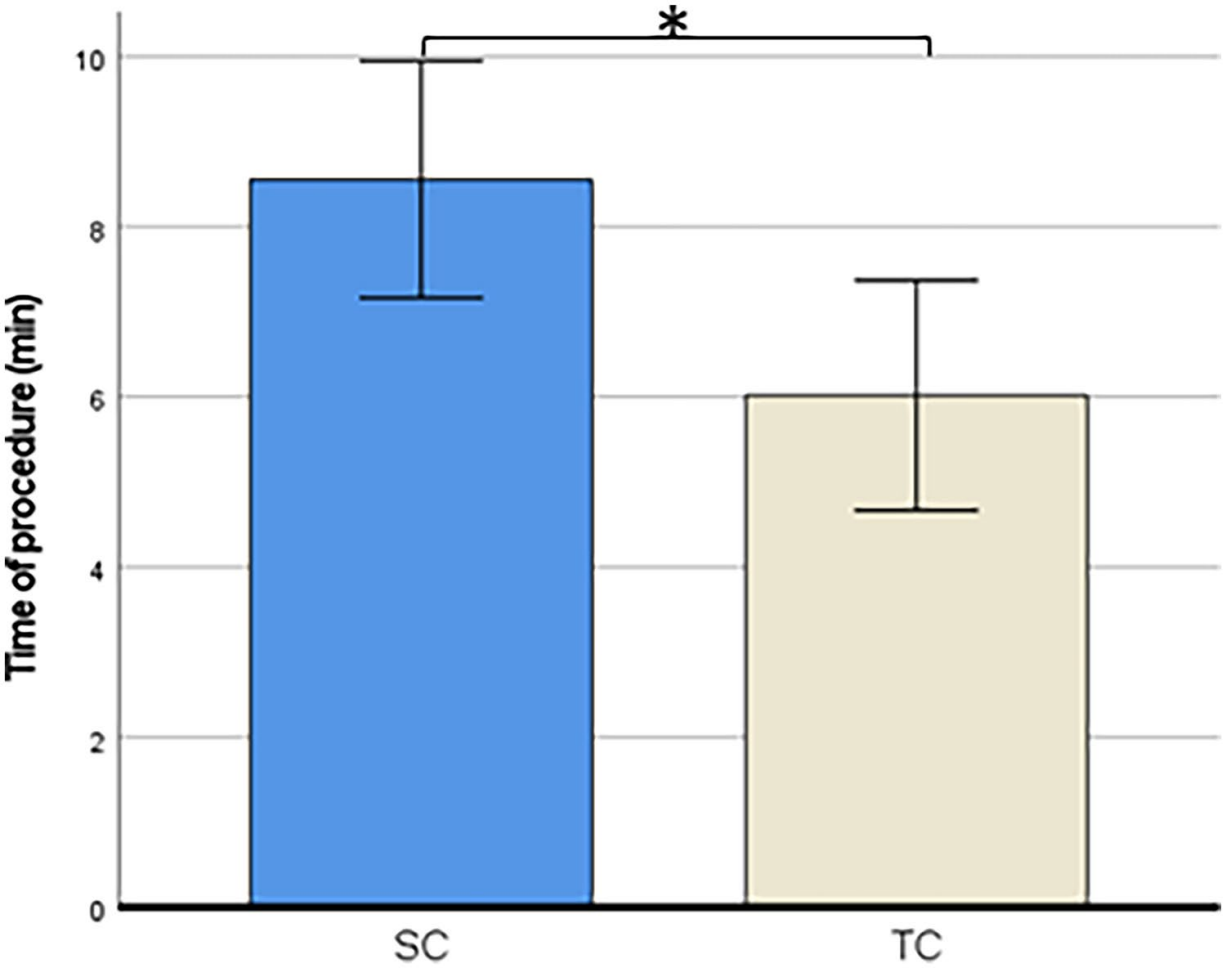
Procedural time. *SC* = *Standard communication, TC* = Therapeutic communication. 95% Error bars, * < *.05*

Procedural time in the TC group was shorter (6min0s) than in the SC group (8min36s), *t*(103) = 2.53, *p* = .014 (Fig. 5).

#### Mediation analyses

**Fig. 6:**
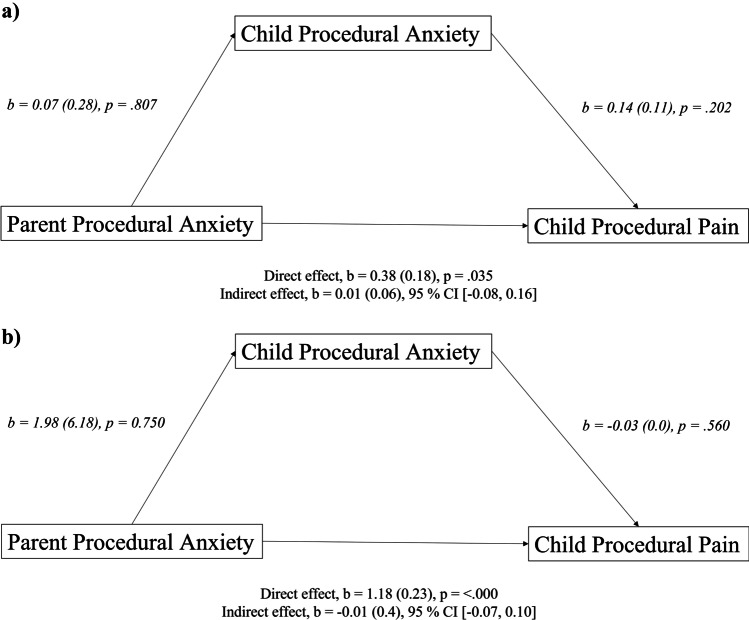
Standardized regression coefficients between parent procedural anxiety, child procedural anxiety and child reported pain for the standard communication group (**a**) and the therapeutic communication group (**b**). Standard errors are in parentheses

Two mediation analyses were conducted to test the direct effect (parental procedural anxiety on child pain) and indirect effects (parental procedural anxiety on child pain via child anxiety) for the TC group and the SC group. The regression models were significant for the SC group (*F*(2,35) = 3.30, *p* = 0.048, *R*^2^ = 0.16) and the TC group (*F*(2,52) = 13.55, *p* < 0.001, *R*^2^ = 0.35). Based on the presence of zero in the confidence intervals for indirect paths, both analyses indicated no mediation effect of parental procedural anxiety on child pain via child anxiety (Fig. 6).

#### Subgroup analysis neurodivergence

Based on the significant outcome of child anxiety, the subgroup analysis was performed with anxiety as outcome. There were no differential effects of TC on anxiety for neurodivergent children versus non-neurodivergent children (i.e., no diagnosis of a psychiatric disorder), *F*(2,70) = .52, *p* = 0.596, ηp^2 =^ .015).

## Discussion

The objective of this study was to examine whether patient-centric TC positively affects children’s comfort during venipuncture. Comfort is defined as a ''transient and dynamic state characterized by ease from pain, emotional and physical distress and an emerging sense of positivity, safety, strength and acceptance of one’s situation that is underpinned and sustained by feeling valued, cared for, confident and accepting treatment. Patients seek to be as comfortable as they can be, under the circumstances of their healthcare interaction'' [26]. When assessing patient’s experience during interventional procedures the word comfort avoids the nocebo effect of the word pain. Unfortunately comfort scores correlate only moderately with pain scores [27]. Pain intensity and distress are considered primary outcomes for evaluating psychological interventions for needle-related procedures in children [2]. Pain and fear are positively associated with each other. Fear can increase the pain experience and fear can increase during acute pain [28]. The interdependence of these two variables made it difficult to decide which primary outcome should be defined. Because of the familiarity and routine measurement of pain scores in children in our hospital, we choose pain intensity as primary outcome measure.

No difference in self-reported pain was found between the SC and the TC group. For the secondary outcomes, however, scores significantly improved for anxiety, time in the room and satisfaction of the medical personnel. For the third research question, namely whether parental anxiety influences the child’s pain via the child’s anxiety, no mediation effect was found. For the fourth research question, we found that TC had the same effect on anxiety as in non-neurodivergent children.

The absence of significant self-reported pain results may be attributed to the overall low pain scores (95% CI [1.1, 2.4]), and these low pain scores did not indicate clinically relevant pain according to the optimal cut points for the FPS-R [29]. The low pain scores are probably the result of the use of EMLA which is an effective local anesthetic for pediatric venipuncture pain [30, 31]. This was implemented as standard procedure prior to venipuncture in our hospital. Literature varies on the effect of therapeutic communication/distraction techniques on pain, because these studies are difficult to compare as they differ in the use of EMLA, distraction technique, patient population and pain scores during venipuncture [10, 32–34].

All anxiety scores for the child (scored by child 8 years or older), parent and medical personnel) were lower in the TC group than in the SC group. The parents also scored lower self-reported NRS anxiety scores when TC was used. NRS anxiety for the parent scored by the medical personnel did not differ significantly between groups. Moreover, this study investigated the influence of parental anxiety on the relationship between the child’s anxiety and pain experience. No mediation effects were found (parental procedural anxiety on child pain via child anxiety) in both groups. This is in contrast with the study results of Bearden et al. (2012) where a mediation effect was found of preprocedural anxiety and procedural pain of the child, which in turn heightened the child’s pain [13]. This, again, may be due to the lack of higher pain scores in the current study. For satisfaction, this study found higher satisfaction for medical personnel in the TC group. We hypothesize that this higher satisfaction of the medical personnel was explained by more communication tools of the personnel to deal with anxious patients and their parents, which resulted in less delay and less stressful situations. Furthermore the personnel mentioned a better team climate and improved interaction between care givers themselves. However not investigated thoroughly in our study, this improved job satisfaction and better team climate was also described in other studies [11, 35]. The shorter procedural time and no need for other resources makes this communication technique very suitable for application in the outpatient clinic and at the emergency department.

 Finally, because a large proportion of patient undergoing venipuncture is neurodivergent (28.6% in our sample), and because of the limited research conducted on this topic, we performed a subgroup analysis to find out if TC was also effective in the neurodivergent children (e.g., with ASD or ADHD) in our study. The results showed similar effects in anxiety reduction for neurodivergent children as non-neurodivergent children. However, this result must be interpreted with caution, as this analysis was exploratory and the sample of the subgroup analysis was relatively small (n = 30).

One of the strengths of this study was that when examining comfort during venipuncture, this study captured the multi-faceted context of this procedure. Different outcomes were measured (e.g., pain, anxiety, and satisfaction) from different perspectives (children, parents and medical personnel) thereby adopting an overall integrative approach. Moreover, this study aimed to contribute to the scarce literature of venipuncture comfort for neurodivergent children. However, adopting this integrative approach induces bias. Given the large amount of outcome measures, the likelihood of incorrectly rejecting a null hypothesis (i.e., making a Type I error) increases [36]. Furthermore, we recommend more emphasis on (neuro)divergent groups in future research, especially for children with ASD as these children often show higher levels of anxiety [19, 37–39].

To conclude, our study found that with just a small change in communication style, the comfort of the child during venipuncture improves. This was mainly reflected by reduced anxiety scores and shorter procedural times, making the use of TC during venipuncture promising for the outpatient setting.

### Supplementary Information

Below is the link to the electronic supplementary material.

Supplementary file1 (PDF 127 KB)

## List of abbreviations

ADHD: Attention deficit hyperactivity disorder
ANOVA: Analysis of variance
ASD: Autism spectrum disorder
EMLA: Eutectic mixture of local anesthetics
FPS-R: Faces pain scale-revised
NRS: Numeric rating scale
SC: Standard communication
STAI-C: State trait anxiety inventory for children
STAI-Y: State trait anxiety inventory for adults (form Y)
TC: Therapeutic communication
ZBV: Zelf beoordelingsvragenlijst (ENG: self-report questionnaire)

## Declarations

### Storage of data

Subject, code and patient identification were saved in an identification log. A key file with password for the identifying personal data, linked to the study code, was saved on a separate file and was only accessible for the researchers. On the Case Report Form and in the database (Castor EDC) only coded data was used, no personal identification data. Data was saved for 15 years.
