# Supplementary material for: Therapeutic communication improves patient comfort during venipuncture in children: a single-blinded intervention study
Source: Eur J Pediatr. 2023 Jun 17;182(9):3871–81. doi: 10.1007/s00431-023-05036-7 (PMC10570224; doi:10.1007/s00431-023-05036-7)
Supplement: Supplementary file 1 — Supplementary file1 (PDF 127 KB) [file 431_2023_5036_MOESM1_ESM.pdf]

Journal: European Journal of Pediatrics

Title: Therapeutic communication improves patient comfort during venipuncture in children: A single-blinded intervention study

Authors: Lonneke AM Aarts, Geert-Jan van Geffen, Eva AL Smedema, Rosanne M Smits.

Corresponding author: Lonneke AM Aarts, MD, Department of Pediatrics, Amalia Children's Hospital, Radboud University Medical Center, Nijmegen, the Netherlands. lonneke.aarts@radboudumc.nl

Supplementary Table 1: An overview of therapeutic communication techniques applied in our study with examples.

**Therapeutic communication techniques**	**Example**
Instant rapport (matching)	Non-verbal matching (eye contact, posture, behavior and breathing rate), verbal matching (speaking volume, tempo, word choice).
Avoid sabotage words/ sentences	Try, don't, always, pain, sharp, hurt, cry, cold, " here it comes'', ''do you think it is exciting?''
Positive suggestion	Just blow out gently and you will notice that this makes you more relaxed.
Use positive words	Help, secure, comfort, warm, together.
Imagery	Where would you rather be at this moment? Or: What would you rather do at this moment? Tell me about your hobbies, holidays, sport, school….
Reversed effect	"You may try to keep your eyes open. "(Try implies failure).
Presumed outcome	"How deep do you want to relax, deep, moderate or extra deep" (You always relax).
Dissociation	I will hold the arm (in stead of " your arm)
Distraction/confusion	" You want me to count before starting? Fourteen, fifteen, sixteen…" (confusion).

Self-experience	Avoid expressing observed emotions. Leave the interpretation of expression to the child. Every child has his own experience. If it is uncomfortable they will tell you.
Double bind	“What do you want, climbing on the table yourself of that mum/dad helps you?
Direct command	“Take a deep breath and blow bubbles: now!’’
Complimenting	Instead of; “well done, you are a big boy/girl’’ say: “because you kept your arm so still, I could easily take blood and that’s why we are done superfast.
